# A Novel Inhibitor of α9α10 Nicotinic Acetylcholine Receptors from *Conus vexillum* Delineates a New Conotoxin Superfamily

**DOI:** 10.1371/journal.pone.0054648

**Published:** 2013-01-30

**Authors:** Sulan Luo, Sean Christensen, Dongting Zhangsun, Yong Wu, Yuanyan Hu, Xiaopeng Zhu, Sandeep Chhabra, Raymond S. Norton, J. Michael McIntosh

**Affiliations:** 1 Key Laboratory of Tropical Biological Resources, Ministry of Education, Key Lab for Marine Drug of Haikou, Hainan University, Haikou Hainan, China; 2 Departments of Biology and Psychiatry, University of Utah, Salt Lake City, Utah, United States of America; 3 Medicinal Chemistry, Monash Institute of Pharmaceutical Sciences, Monash University, Parkville, Australia; National Research Council of Italy, Italy

## Abstract

Conotoxins (CTxs) selectively target a range of ion channels and receptors, making them widely used tools for probing nervous system function. Conotoxins have been previously grouped into superfamilies according to signal sequence and into families based on their cysteine framework and biological target. Here we describe the cloning and characterization of a new conotoxin, from *Conus vexillum*, named αB-conotoxin VxXXIVA. The peptide does not belong to any previously described conotoxin superfamily and its arrangement of Cys residues is unique among conopeptides. Moreover, in contrast to previously characterized conopeptide toxins, which are expressed initially as prepropeptide precursors with a signal sequence, a ‘‘pro’’ region, and the toxin-encoding region, the precursor sequence of αB-VxXXIVA lacks a ‘‘pro’’ region. The predicted 40-residue mature peptide, which contains four Cys, was synthesized in each of the three possible disulfide arrangements. Investigation of the mechanism of action of αB-VxXXIVA revealed that the peptide is a nicotinic acetylcholine receptor (nAChR) antagonist with greatest potency against the α9α10 subtype. ^1^H nuclear magnetic resonance (NMR) spectra indicated that all three αB-VxXXIVA isomers were poorly structured in aqueous solution. This was consistent with circular dichroism (CD) results which showed that the peptides were unstructured in buffer, but adopted partially helical conformations in aqueous trifluoroethanol (TFE) solution. The α9α10 nAChR is an important target for the development of analgesics and cancer chemotherapeutics, and αB-VxXXIVA represents a novel ligand with which to probe the structure and function of this protein.

## Introduction

Nicotinic acetylcholine receptors (nAChRs) are pentameric ligand-gated ion channels used throughout the animal kingdom for neurotransmission. These receptors are assembled from α,β,γ,δ and/or ε subunits to form multiple receptor subtypes with distinct pharmacological properties [1]. Elucidation of the precise structure and function of various nAChRs is challenging owing to the scarcity of ligands selective for specific receptor subtypes. In an effort to address this, we have systematically examined components of the venoms of carnivorous cone snails for selective nAChR-targeted ligands.

Molluscs of the genus *Conus* are comprised of >700 species. These marine snails hunt primarily polychaete worms, molluscs or fish. Each cone species produces a cocktail of >100 different compounds that enables prey capture. Despite extensive work, the vast majority of these compounds remains uncharacterized. Conopeptides are produced in the venom duct of *Conus* and used offensively to immobilize prey. Their potency and selectivity for various ion channels and receptors have made them excellent pharmacological probes and drug leads [2]–[3]. The term conotoxin is used to describe the subset of *Conus* peptides that are rich in Cys residues. Conotoxins are synthesized initially as precursor proteins that are subsequently processed into the mature toxin. Previously characterized *Conus* peptides have been grouped into gene superfamilies based on similarities in their precursor signal sequences [4]. Within each superfamily, the toxins are grouped according to cysteine frameworks that influence their final three-dimensional structure. The toxins are also grouped according to receptor or ion channel target into pharmacological families. Within a given family of conotoxins there is, characteristically, hypervariation in non-Cys residues, which is believed to enable selective action on a given target subtype. Post-translational modification or chemical synthetic modification provides further diversity [5]–[6].

Toxins characterized to date can be classified into one of 17 superfamilies (see Table 1) [7]–[8]. The current study characterizes a new conotoxin, from the worm-hunting *Conus vexillum*, with a unique Cys framework. As the precursor sequence does not align with any of the previously-reported gene superfamilies, this peptide represents a first-in-class compound (see (Table S1)). Total chemical synthesis was carried out to enable pharmacological and structural characterization of this novel toxin. The peptide acts as an antagonist of nicotinic acetylcholine receptors, with greatest potency at the α9α10 nAChR, a subtype expressed in a variety of tissues ranging from immune cells to sperm [9]–[10].

**Table 1:** Protein precursor sequences of *Conus* gene superfamilies [46]–[61].

Super-family	Peptide	Precursor Sequence (Signal, *N-terminal pro-regions,* ↓Mature peptide and ↓*C-terminal pro-regions*)	Reference
B	αB-VxXXIVA	METLTLLWRASSSCLLVVLSHSLLRLLG↓VR**C**LEKSGAQPNKLFRPP**CC**QKGPSFARHSR**C**VYYTQSRE	This Study
A	α-LtIA	MGMRMMFIMFMLVVLATTVVTFTS*DRALDAMNAAASNKA* *SRLIALAVR*↓G**CC**ARAA**C**AGIHQEL**C**↓GGGR	46,47,48
D	αD-VxXXB	MPKLAVVLLVLLILPLSYFDAAGG*QAVQGDWRGNRLARD* *LQRGGR*↓DDESE**C**IINTRDSPWGR**CC**RTRM**C**GSM**CC**PRNG**C**T**C**VYHWRRGHG**C**S**C**PG	49,50
I_1_	ArXIA	MKLCATFLLVLVTLPLVTG*EKSSERSLSGAILRGVR*↓RT**C**S RRGHR**C**IRDSQ**CC**GGM**CC**QGNR**C**FVAIRR**C**FHLPF	51
I_2_	BeTX	MMFRVTSVGCLLLVIVFLNLVVPTSA↓**C** RAEGTY**C**ENDS Q**CC**LNE**CC**WGG**C**GHP**C**RHP↓*GKRSKLQEFFRQR*	52
I_3_	Ca11.3	MKLVLAIVVILMLLSLSTGA*EMSDNHASRSATALRDRLLSP* *K*↓ASI**C**YGTGGR**C**TKDKH**CC**GWL**CC**GGPSVG**C**VVSVAP**C**K	53
J	Fe14.1	MPSVRSVTCCCLLWMMLSVQLVTPGSPGTAQLSGHRTAR↓SPGSTI**C**KMA**C**RTGNGHKYPFCNCR↓GKRDVVSSSMAV	31
K	im23a	MIMRMTLTLFVLVVMTAASASG*DALTEAKR* IPYCGQTGA ECYSWCIKQDLSKDWCCDFVKDIRMNPPADKCP	8
L	C14.1a	MNVTVMFLVLLLLTMPLTDG*FNIRATNGGELFGPVQRDAG* *NVLDHGFQRRR*↓D**C**PPW**C**PTSH**C**NAGT**C**	54
M	ψ-PrIIIE	MSKLGVLLTICLLLFPITA*LPVDGDQPADRPVERMQDNISS* *EQHPFFEKR*↓AAR**CC**TYHGS**C**LKEK**C**RRKY**CC**↓GR	55
O_1_	SO3	MKLTCMVIVAVLLLTACQLITA*DDSRGTQKHRTLRSKTKL* *SMSTR*↓**C** KAAGKP**C**SRIAYN**CC**TGS**C**RSGK**C**↓G	56
O_2_	BeB54	MEKLTILLLVAAVLMSTQALI*QSDGEKRQQAKINFLS.R*↓K STAESWWEGE**C**KGWSVY**C**SWDWE**CC**SGE**C**TRYY**C**ELW	17
O_3_	CaFr179	MSGLGIMVLTLLLLVFMEA*SHQDAGEKQATQRDAINVRR* *RRSLARR*↓TVTEE**C**EED**C**EDEEKH**CC**NTNNGPS**C**ARL**C**F↓G	17
P	GmIXA	MHLSLARSAVLMLLLLFALGNFVVVQS*GLITRDVDNGQL* *TDNRRNLQTEWNPLSLFMSRR*↓S**C**NNS**C**QSHSD**C**ASH**C**I**C**TFRG **C**GAVN↓G	57
S	αS-GVIIIA	MMSKMGAMFVLLLLFTLAS*SLQEGDVQARKTRLKSDFYR* *ALARDDR*↓G**C**TRT**C**GGPK**C**TGT**C**T**C**TNSSK**C**G**C**RYNVHPSGWG**C**G**C**A**C**S↓G	58
T	VcVB	VILLLLIASAPSVDA*QPKTKDDVPLAPLHDNAKSALQHLNQ* *R*↓**CC** QTFYW**CC**GQ↓GK	59
V	ViXVA	MMPVILLLLLSLAIRCADG*KAVQGDSDPSASLLTGDKNHD* *LPVKR*↓D**C**TT**C**AGEE**CC**GR**C**T**C**PWGDN**C**S**C**IEW↓GK	60
Y	CaXVIIA	MQKATVLLLALLLLLPLSTA*QDAEGSQEDAAQREVDIATR*↓**C** GGTGDS**C**NEPAGEL**CC**RRLK**C**VNSR**CC**PTTDG**C**	61

## Materials and Methods

### Ethics Statement

No specific permits were required for the described field studies. No specific permissions were required for Tanmen Qionghai, Hainan Province, China, which is not privately-owned or protected in any way. The field studies did not involve endangered or protected species.

### Materials

Specimens of *Conus vexillum* were collected from the South China Sea off Tanmen Qionghai, Hainan Province, China. Venom ducts were frozen and stored at −80°C. Creator SMART cDNA Library Construction Kit was from CLONTECH Laboratories, Inc. Acetylcholine chloride, atropine, and bovine serum albumin (BSA) were from Sigma. The reverse-phase HPLC analytical Vydac C18 column (5 µm, 4.6 mm×250 mm) and preparative C18 Vydac column (10 µm, 22 mm×250 mm) were from Shenyue. Reagents for peptide synthesis were from GL Biochem. Acetonitrile was from Fisher. Trifluoroacetic acid (TFA) was from Tedia. All other chemicals used were of analytical grade. Clones of rat α2–α7 and β2–β4, as well as mouse muscle α1β1δε cDNAs were kindly provided by S. Heinemann (Salk Institute, San Diego, CA). Clones for α9 and α10 were generously provided by A.B. Elgoyen (Instituto de Investigaciones en Ingeniería Genética y Biología Molecular, Buenos Aires, Argentina). Clones of β2 and β3 subunits in the high expressing pGEMHE vector were kindly provided by C.W. Luetje (University of Miami, Miami, FL).

### cDNA Sequencing

Total RNA was extracted from individual ducts and purified as described previously [11]. Venom duct cDNA library construction followed the kit manufacturer’s suggested protocol. Briefly, the first-strand cDNA was synthesized with the SMART IV Oligonucleotide and transcriptase. Full-length, double-stranded (ds) cDNA (SMART cDNA) was generated by long-distance PCR. SMART cDNA was ligated into the Sfi I predigested pDNR-LIB vector. The signal and mature peptide sequences of the conotoxin precursors were predicted using online ProP 1.0 Server [12].

### Peptide Synthesis

The linear peptide was assembled by solid-phase methodology on an ABI 433A peptide synthesizer using FastMoc (N-(9-fluorenyl) methoxycarbonyl) chemistry and standard side-chain protection, except for cysteine residues. Cys residues of the three possible isomers were protected in pairs with either S-trityl on Cys3 and Cys19 (designated αB-VxXXIVA [1], [2]), Cys3 and Cys20 (designated αB-VxXXIVA [1], [3]), Cys19 and Cys20 (designated αB-VxXXIVA [1], [4]) or S-acetamidomethyl on Cys20 and Cys32, Cys19 and Cys32, Cys3 and Cys32, respectively. The peptides were removed from a solid support by treatment with reagent K (TFA / water / ethanedithiol / phenol / thioanisole; 90∶ 5 : 2.5∶ 7.5∶ 5,v / v / v / v / v). The released peptide was precipitated and washed three times with cold ether. A two-step oxidation protocol was used to fold the peptides selectively, as described previously [13]. Briefly, the disulfide bridge between Cys3 and Cys19, Cys3 and Cys20, or Cys19 and Cys20, respectively, was closed by dripping the peptide into an equal volume of 20 mM potassium ferricyanide, 0.1 M Tris, pH 7.5. The solution was allowed to react for 45 min, and the monocyclic peptide was purified by reverse-phase HPLC. Simultaneous removal of the S-acetamidomethyl groups and closure of the disulfide bridge between Cys20 and Cys32, Cys19 and Cys32, or Cys3 and Cys32, respectively, was carried out by iodine oxidation as follows: the monocyclic peptide in HPLC eluent was dripped into an equal volume of iodine (10 mM) in H_2_O:TFA:acetonitrile (74∶2:24 by volume) and allowed to react for 10 min. The reaction was terminated by the addition of ascorbic acid, diluted 10-fold with 0.1% TFA, and the bicyclic peptide was purified by HPLC on a reversed-phase C18 Vydac column using a linear gradient of 20–60% B60 in 40 min. Solvent B was 60% ACN, 0.092% TFA, and H_2_O; Solvent A 0.1% TFA in H_2_O. Peptide concentration was measured using absorbance at 280 nm, and calculated using the Beer-Lambert equation and a calculated molar extinction coefficient of 3040 cm^−1^ M^−1^.

### cRNA Preparation and Injection

Capped cRNA for the various subunits were made using the mMessage mMachine *in vitro* transcription kit (Ambion) following linearization of the plasmid. The cRNA was purified using the Qiagen RNeasy kit. The concentration of cRNA was determined by absorbance at 260 nm. Oocytes were injected within one day of harvesting and recordings were made 1–4 days post-injection.

### Voltage-clamp Recording

Oocytes were voltage-clamped and exposed to ACh and peptide as described previously [14]. Briefly, the oocyte chamber consisting of a cylindrical well (∼30 µl in volume) was gravity perfused at a rate of ∼2 ml/min with ND96 buffer (96.0 mM NaCl, 2.0 mM KCl, 1.8 mM CaCl_2_, 1.0 mM MgCl_2_, 5 mM HEPES, pH 7.1–7.5) containing 0.1 mg/ml BSA. The Ba^++^-ND96 had 1.8 mM BaCl_2_ in place of CaCl_2_. The membrane potential of the oocytes was clamped at −70 mV. The oocyte was subjected once a minute to a 1 s pulse of 100 µM ACh. In the case of the α9α10 and muscle α1β1δε subtypes, there is a 1 s pulse of 10 µM Ach, and for the α7 subtype a 200 µM ACh pulse. For toxin concentrations ≥10 µM, once a stable baseline was achieved, either ND-96 alone or ND-96 containing conotoxin was applied manually for 5 min prior to the addition of the agonist. All recordings were done at room temperature (∼22°C).

### Data Analysis

The average of five control responses just preceding a test response was used to normalize the test response to obtain “% response.” Each data point of a dose-response curve represents the average ± S.E. of at least three oocytes. The dose-response data were fit to the equation, % response = 100/[1+ ([toxin]/IC50)?n_H_], where n_H_ is the Hill coefficient, by non-linear regression analysis using GraphPad Prism (GraphPad Software).

### NMR Spectroscopy

^1^H NMR spectra were recorded on αB-VxXXIVA isomers at a concentration of ∼ 350 µM in 20 mM phosphate/10% ^2^H_2_O buffer at pH ∼ 5.8. The 1D spectra were acquired on a Bruker Avance 600 MHz NMR spectrometer equipped with cryogenic probe fitted with a *z* axis gradient. The NMR spectra were collected at 5°C using the excitation sculpting pulse sequence [15]. Spectra were acquired over 4K data points with 64 scans and a ^1^H spectral width of 14 ppm. All spectra were processed in TOPSPIN (version 3.0) and referenced to the water resonance.

### Circular Dichroism Analysis

αB-VxXXIVA isomers were dissolved in 20 mM phosphate buffer (pH 5.9) and CD spectra were recorded on a Jasco-815 spectropolarimeter at a concentration of 43 µM at 20°C. Spectra were collected at 0.05 nm intervals over the wavelength range 260–195 nm in a 10 mm pathlength cuvette. Three scans were collected and averaged for each peptide sample with scanning rate of 100 nm/min^−1^. The spectra were then smoothed using a third-order polynomial function. In order to investigate the effect of trifluoroethanol (TFE) on the conformation, CD spectra for αB-VxXXIVA [1], [2] were also acquired following the addition of 10, 20, 50 and 87% TFE. The % α-helix and β-sheet content were calculated from the CD data using the CDPro program [16].

## Results

### Discovery and Sequence Analysis of a cDNA of the Precursor of αB-VxXXIVA

**Figure 1:**
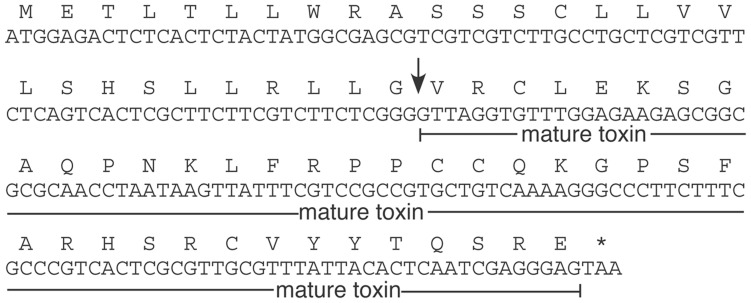
αB-Conotoxin VxXXIVA prepeptide and encoded toxin are shown. A putative proteolytic processing site (G) is indicated by the arrow. The mature toxin region is underlined. The stop codon is indicated as *. Unlike previously reported conotoxins, there is no pro region.

In general, conotoxins are translated initially as prepropeptide precursors [7], with proteolytic cleavage yielding the final product(s). Peptides in the same superfamily are characterized by highly conserved prepropeptide precursor sequences. This conservation has allowed direct identification of new peptides belonging to a particular superfamily by cDNA sequencing of family or superfamily genes [17]. However, a large fraction of conotoxins present in the *Conus* genus has yet to be sequenced and several additional families of toxins remain to be identified. Most of the cone snails investigated to date are fish- or mollusc-hunters. In an effort to discover novel conotoxin families, we examined the worm-hunting *C. vexillum.* Specimens were collected from the South China Sea and dissected venom ducts were used to construct a cDNA library. Approximately 50 clones from the cDNA library were chosen randomly for sequencing and inspected for previously unreported sequences. In the present study with *C. vexillum*, several members of the previously characterized α- and ω- superfamilies were identified. In addition, however, an unusual precursor sequence was noted (Fig. 1, Table 1, GenBank accession number JX297421). A sequence similarity search detected no homology with precursors of the known superfamilies of conotoxins [7] (Table S1). The sequence was analyzed with DNAstar software and online ProP 1.0 Server [12], which indicated a 28-residue signal sequence followed by a previously unreported 40-residue mature toxin (Table 2 and see also Table S2 for sequence alignment). For other conotoxins, the encoding cDNA has a characteristic three-region organization, including a signal sequence, a ‘‘pro’’ region, and the toxin-encoding region [7], [18] The generation of the mature toxin requires proteolytic cleavage of the N-terminal prepro-region of the precursor. In contrast to previously characterized conopeptide toxins, the precursor of αB-VxXXIVA has no ‘‘pro’’ region. The putative proteolytic processing site between prepropeptide and mature region for conotoxins is usually a basic amino acid (K or R). In contrast, the predicted cleavage site for the αB-VxXXIVA precursor is –LG- (Fig. 1). The predicted mature peptide exhibited a new cysteine framework, not previously reported for conotoxins, C–CC–C (Table 2 and Table S2). The predicted mature toxin sequence was VRCLEKSGAQPNKLFRPPCCQKGPSFARHSRCVYYTQSRE.

**Table 2:** Mature toxin sequences of nAChR-targeted conotoxin superfamilies [62]–[68].

Peptide	Superfamily	Mass	Cysteine Residues	Sequence	Reference
αB-VxXXIVA	B	4623	4	VR**C**LEKSGAQPNKLFRPP**CC**QKGPSFARHSR**C**VYYTQSRÊ 40aa	This Study
α-AuIB	A	1573	4	G**CC**SYPP**C**FATNPD**C**# 15aa	62
αA-OIVB	A	1865	6	**CC**GVONAA**C**PO**C**V**C**NKT**C**G# 19aa	63
αC-PrXA	T	3492	2	TYGIYDAKPOFS**C**AGLRGG**C**VLPONLROKFKE# 32aa	64
αD-VxXIIA	D	5134	10	DVQD**C**QVSTOGSKWGR**CC**LNRV**C**GPM**CC**PASH**C**Y**C**VYHRGRGHG**C**S**C**? 47aa	65,66
αS-RVIIIA	S	5168	10	K**C**NFDK**C**KGTGVYN**C**GXS**C**S**C**XGLHS**C**R**C**TYNIGSMKSG**C**A**C**I**C**TYŶ 47aa	67
ψ-PIIIE	M	2716	6	HOO**CC**LYGK**C**RRYOG**C**SSAS**CC**QR# 24aa	68

O = hydroxyproline, X = gamma-carboxyglutamate, # = C-terminus amidation,

∧ = C-terminus COOH.

### Chemical Synthesis and Oxidative Folding of VxXXXIVA

**Figure 2:**
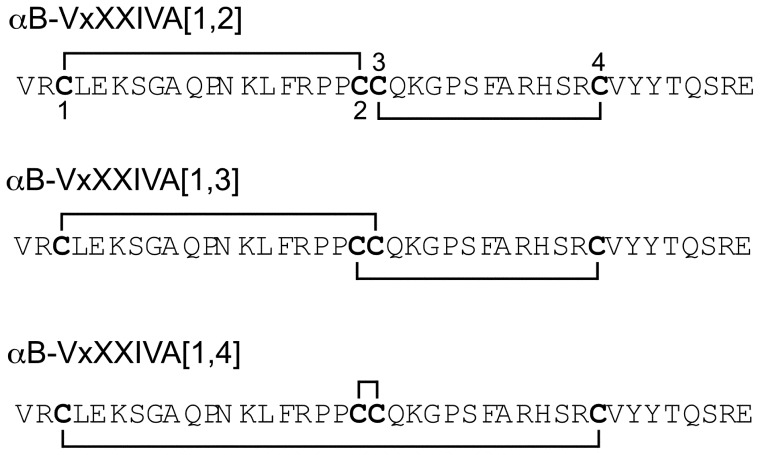
Amino acid sequence of αB-Conotoxin VxXXIVA. Three possible isomers with different disulfide connectivities: αB-VxXXIVA [1], [2] with a disulfide connectivity I–II, III–IV; αB-VxXXIVA [1], [3] with I–III, II–IV and αB-VxXXIVA [1], [4] with I–IV, II–III.

With four Cys residues there are three possible disulfide bond arrangements:Cys3-Cys19, Cys20-Cys32 (αB-VxXXIVA [1], [2]); Cys3-Cys20, Cys19-Cys32 (αB-VxXXIVA [1], [3]), and Cys3-Cys32, Cys19-Cys20 αB-VxXXIVA [1], [4] (Fig. 2). Fmoc chemistry was used to synthesize the linear αB-VxXXIVA peptides. The cysteine side chains were protected in pairs with orthogonal protecting groups that could be removed selectively under different conditions, allowing the formation of one disulfide bridge at a time. The first and second, first and third, or second and third cysteine residues were protected with acid-labile groups, which were simultaneously removed during cleavage from the resin. Ferricyanide was used to close the first disulfide bridge. Reverse-phase HPLC was used to purify the monocyclic peptide; subsequently, the acid-stable acetometomethyl groups were removed from the remaining two cysteines by iodine oxidation, which also closed the second disulfide bridge. The three fully folded peptide isomers were individually purified by HPLC. Electrospray mass spectrometry was utilized to confirm the identity of the products. The monoisotopic masses in Da were: calculated, 4622.27; observed 4622.3 (αB-VxXXIVA [1], [2]), 4622.2 (αB-VxXXIVA [1], [3]), and 4622.4 (αB-VxXXIVA [1], [4]). Synthetic peptides with these disulfide bond arrangements were used in all subsequent studies.

### Effect of Conotoxin αB-VxXXIVA on nAChR ACh-evoked Currents

**Figure 3:**
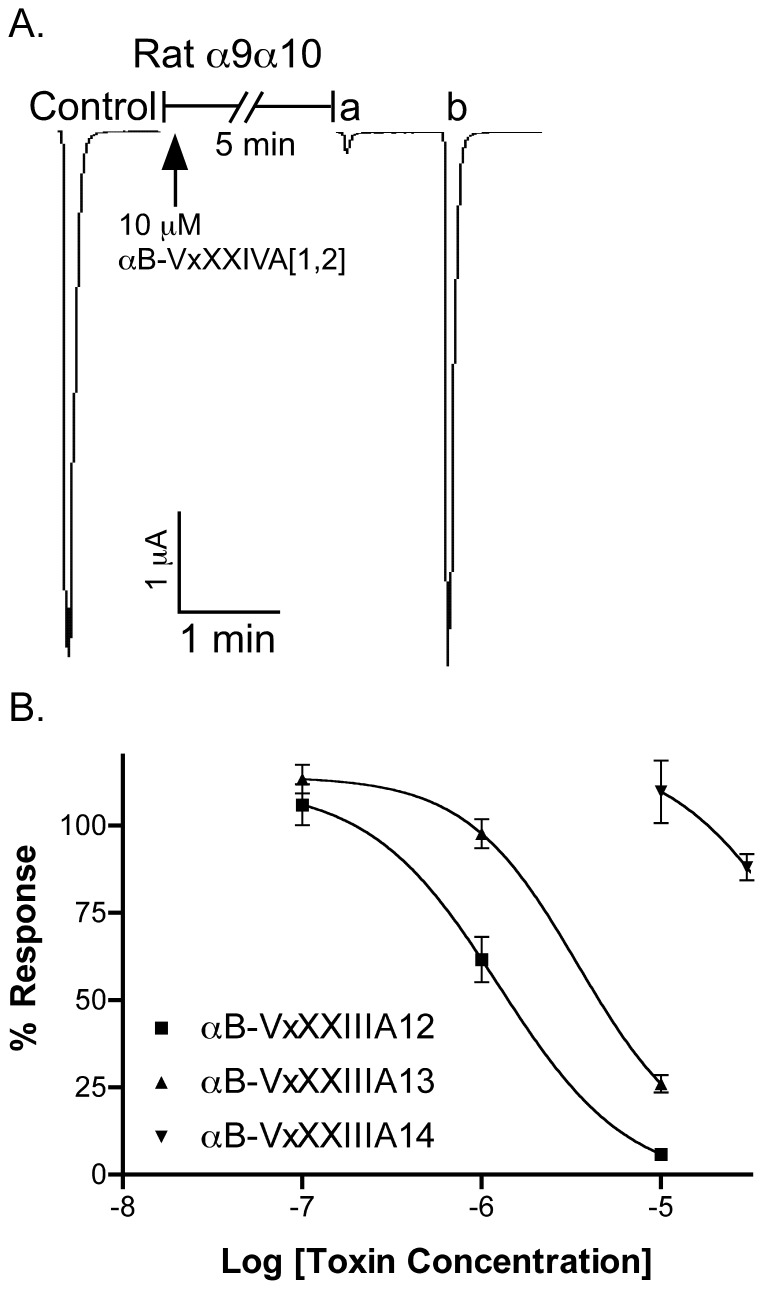
αB-Conotoxin VxXXIVA blocks α9α10 nAChRs. (**A**) *Xenopus* oocytes expressing α9α10 nAChR were voltage clamped at –70 mV and subjected to a 1 s pulse of ACh every min as described in *Materials and Methods*. A representative response in a single oocyte is shown. After control responses to ACh, the oocyte was exposed to 10 µM toxin for 5 min (arrow). After the 5 min toxin application, a response to ACh was measured (a). After 1 min of toxin washout, another response to ACh was measured (b). Note that the response to ACh recovered to control level after 1 min of toxin washout. (**B**) Concentration response of α9α10 nAChRs exposed to the three different isomers of αB-VxXXIVA (see Fig 2). Values shown in the graph are mean ± SEM from 3–5 separate oocytes. The IC_50_s were: αB-VxXXIVA [1], [2], 1.2 µM (0.8–1.7 µM); αB-VxXXIVA [1], [3], 3.9 µM (2.7–5.6 µM); and αB-VxXXIVA [1], [4] >30 µM. Hill slopes were αB-VxXXIVA [1], [2], 1.4 (0.5–2.1) and αB-VxXXIVA [1], [3], 1.3(0.9–1.7).

**Figure 4:**
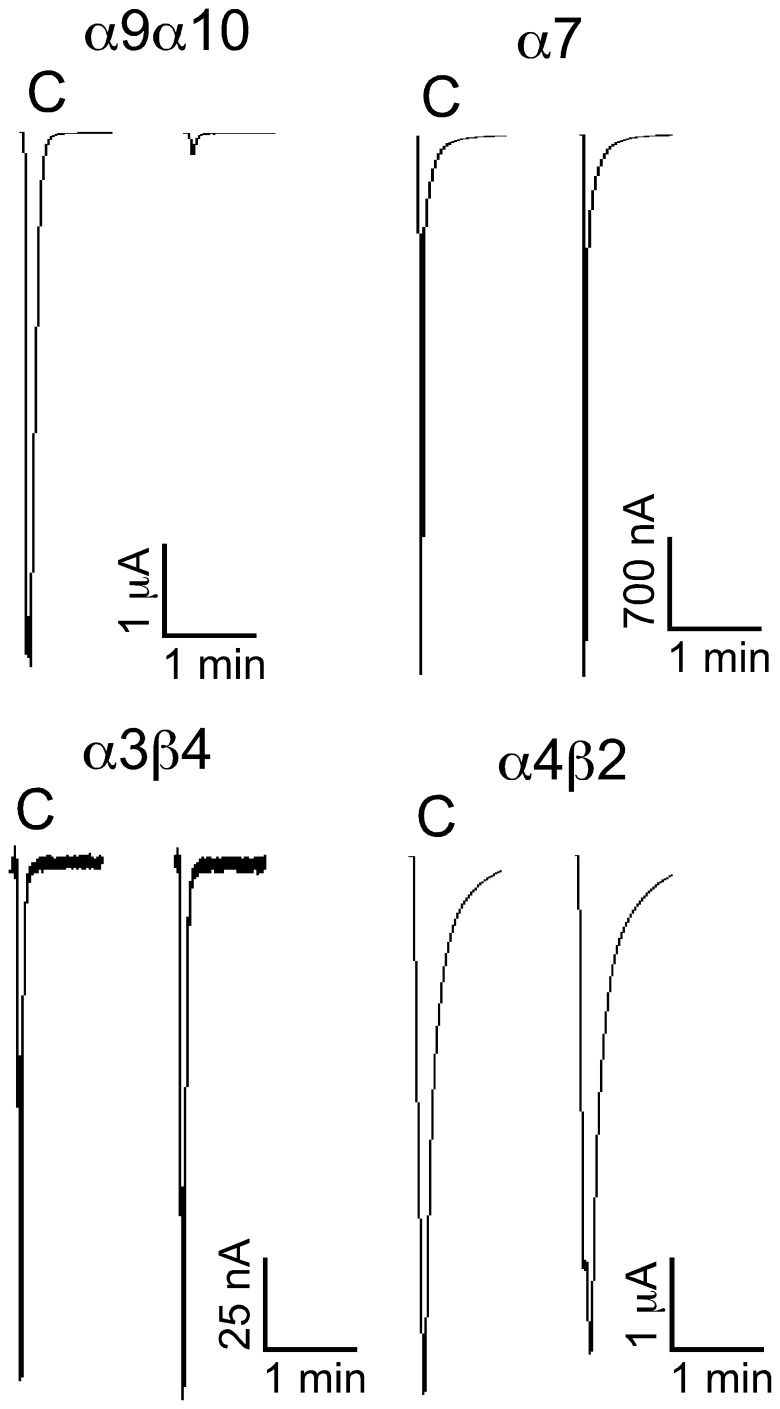
αB-Conotoxin VxXXIVA differentially blocks α9α10, α7, α3β4 and α4β2 nAChRs. nAChR subtypes were expressed as described in *Materials and Methods*. “C” indicates control responses to ACh. Oocytes were then exposed to 10 µM peptide for 5 min, followed by application of ACh. The peptide blocked α9α10 but not α**7,** α3β4 or α4β2 nAChRs.

ACh is used in neurotransmission in the prey of *Conus* and nAChR antagonists are prevalent components of cone snail venoms. We therefore tested the αB-VxXXIVA isomers on subtypes of nAChRs. Pairwise combinations of nAChR subunits were heterologously expressed in *Xenopus* oocytes (Table 3). The toxins were individually tested on these subtypes for their ability to antagonize the response elicited by ACh. Screening was performed initially at 10 µM concentration. Fig. 3A shows representative responses to ACh of α9α10 nAChRs in the presence and absence of αB-VxXXIVA [1], [2]. The block of α9α10 nAChR by αB-VxXXIVA [1], [2] was rapidly reversible. The most potent activity was observed at the α9α10 nAChR subtype (Fig. 3B). Concentration response experiments were then conducted. The IC_50_ of αB-VxXXIVA [1], [2] at the α9α10 nAChR subtype was 1.2 (0.8–1.7) µM. The concentration response for αB-VxXXIVA [1], [2] was subsequently assessed on each of the other expressed nAChR subtypes, Fig. 4 and Table 3. Fig. 4 shows representative responses to ACh of α9α10, α7, α3β4 and α4β2 nAChRs in the presence and absence of αB-VxXXIVA [1], [2]. αB-VxXXIVA [1], [3] was less potent than αB-VxXXIVA [1], [2] on α9α10 nAChRs with an IC_50_ of 3.9 (2.7–5.6) µM. Like αB-VxXXIVA [1], [2], αB-VxXXIVA [1], [3] had little or no activity on other tested nAChR subtypes (IC_50_>30 µM for α2β2, α2β4,α3β2,α3β4,α4β2, α4β4,α6/α3β2β3,α6/α3β4 and α7 nAChRs). αB-VxXXIVA [1], [4] blocked less than 15% of the α9α10 current at the highest concentration tested (30 µM). Likewise, it was inactive at other major nAChR subtypes including α3β4,α4β2 and α7 nAChRs (IC_50_>30 µM).

**Table 3:** IC50 and Hill slope values for block of rat nAChR subtypes by αB-Conotoxin VxXXIVA [1], [2].

nAChR subtype	IC_50_	IC_50_ C.I.	Hill Slope	Hill Slope C.I.
Rat α2β2	23.4 µM	17.3–31.5 µM	1.1	0.7–1.5
Rat α2β4	>30 µM	–	–	–
Rat α3β2	>30 µM	–	–	–
Rat α3β4	>30 µM	–	–	–
Rat α4β2	>30 µM	–	–	–
Rat α4β4	>30 µM	–	–	–
Rat α6/α3β2β3	12.2 µM	10.0–14.9 µM	1.2	0.9–1.7
Rat α6/α3β4	30.1 µM	18.8–48.0 µM	1.0	0.5–1.5
Rat α7	>30 µM	–	–	–
Rat α9α10	1.2 µM	0.8–1.7 µM	1.4	0.5–2.1
Mouse α1β1γδ	6.6 µM	5.1–8.5 µM	1.2	0.8–1.6

C.I., 95% confidence interval.

**Figure 5:**
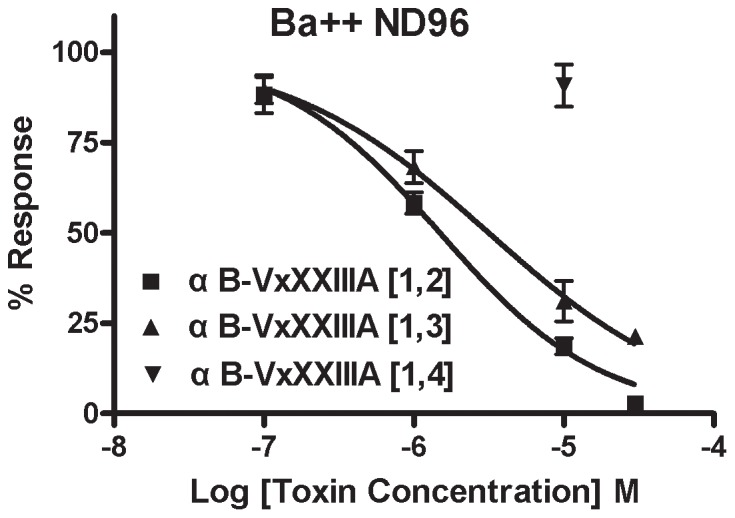
Concentration-response of αB-Conotoxin VxXXIVA on α9α10 nAChR in the presence of Ba^++^. Equimolar Ba^++^ was substituted for Ca^++^, in the perfusion solution as described in *Materials and Methods*, to prevent activation of endogenous *Xenopus* Ca^++^ activated Cl^−^ currents. Values are mean ± SEM from 3–5 separate oocytes. The IC_50_ for the αB-VxXXIVA isomer with disulfide connectivity of I-II; III-IV was 1.49 µM (1.18–1.88) with Hill slope of 0.81 (0.66–0.96). The IC_50_ for the αB-VxXXIVA isomer with disulfide connectivity of I-III; II-IV was 3.15 µM (2.08–4.78) with Hill slope of 0.64 (0.46–0.81). Hill slopes (n_H_) were: Data points shown are the mean ± SEM.

α9α10 nAChRs are known to be highly permeable to calcium. Entry of Ca^++^ through the nAChR elicits a response by Ca^++^-activated chloride currents. The magnitude of this response in *Xenopus* oocytes is large and can comprise >90% of the observed current. In contrast, the closely-related divalent cation Ba^++^ does not elicit a response. We therefore assessed whetherαB-VxXXIVA blocked the response to ACh when Ba^++^ was substituted for Ca^++^ in the buffer. Consistent with previous observations, the ACh response of α9α10 nAChRS in Ba^++^ ND96 was substantially smaller than that observed in Ca^++^ ND96 (data not shown). Using Ba^++^ ND96, the α9α10 nAChR was most potently blocked by αB-VxXXIVA [1], [2] with an IC_50_ of 1.49 µM; under these conditions, αB-VxXXIVA [1], [3] had an IC_50_ of 3.15 µM and αB-VxXXIVA [1], [4] did not potently block the α9α10 nAChR subtype (Fig. 5). Thus, the potency of the αB-VxXXIVA isomers in the presence of Ba^++^ was similar to that seen in Ca^++^, consistent with the toxin effect being due to blockade of the nAChR rather than blockade of the Ca^++^-activated Cl^−^ channel.

### NMR Studies

**Figure 6:**
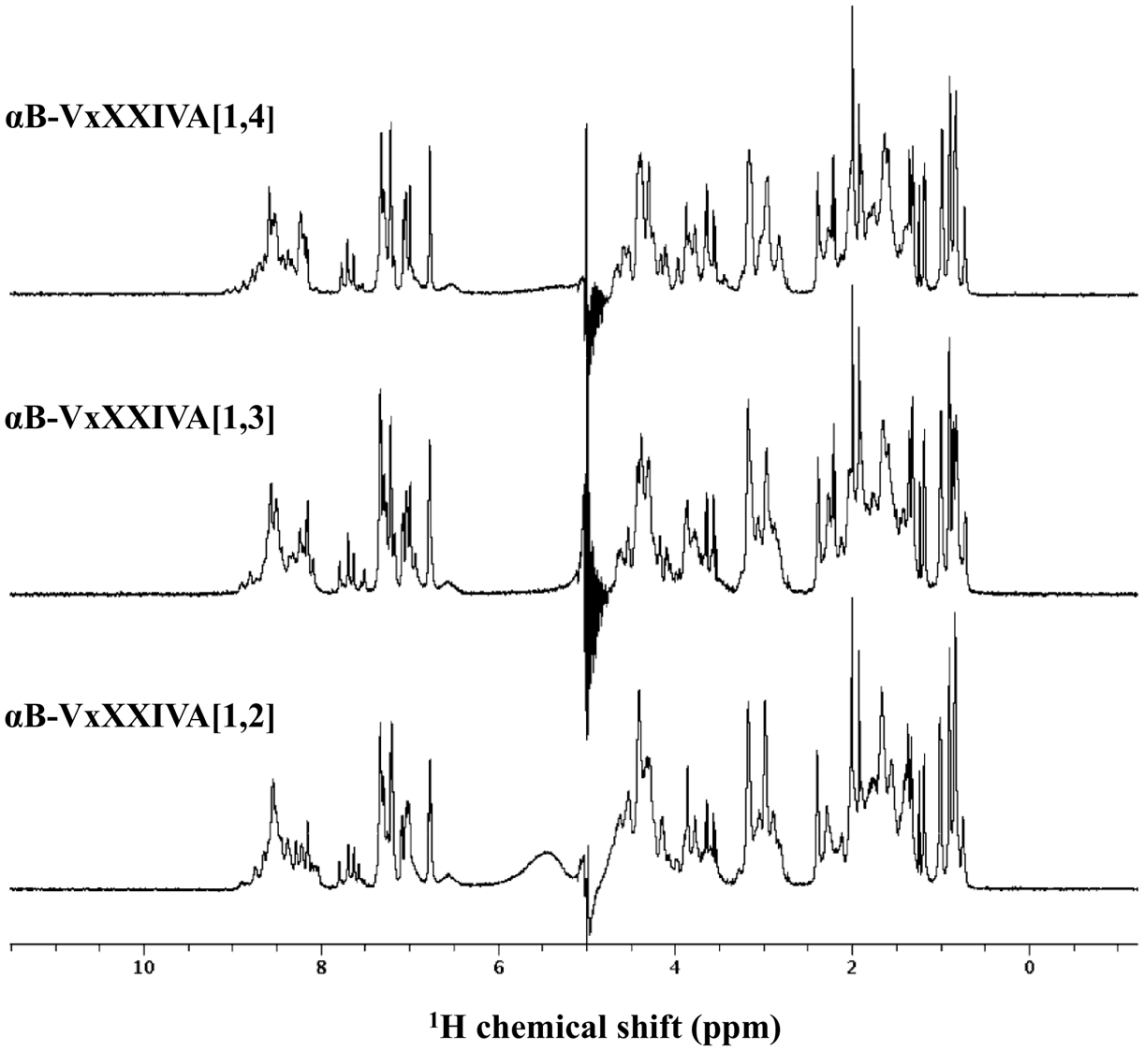
^1^H NMR spectra of αB-Conotoxin VxXXIVA isomers. Peptides were dissolved in 20 mM phosphate buffer at pH 5.8 and spectra were acquired at 600 MHz.

The 1D ^1^H NMR spectra of αB-VxXXIVA isomers in phosphate buffer at pH 5.8 show that the majority of the amide protons fall within the 8.0–9.0 ppm range (Fig. 6); the lack of chemical shift dispersion here and elsewhere in the spectrum indicates that these isomers lack any significant tertiary structure. The same was true at pH 7.0 (Fig. S1, S2, S3). NMR spectra were also acquired in the presence of 3–10 mM CaCl_2_ to ascertain whether calcium had any effect on their conformation, but no change in chemical shift dispersion was observed (Fig. S4).

### Circular Dichroism Analysis

**Figure 7:**
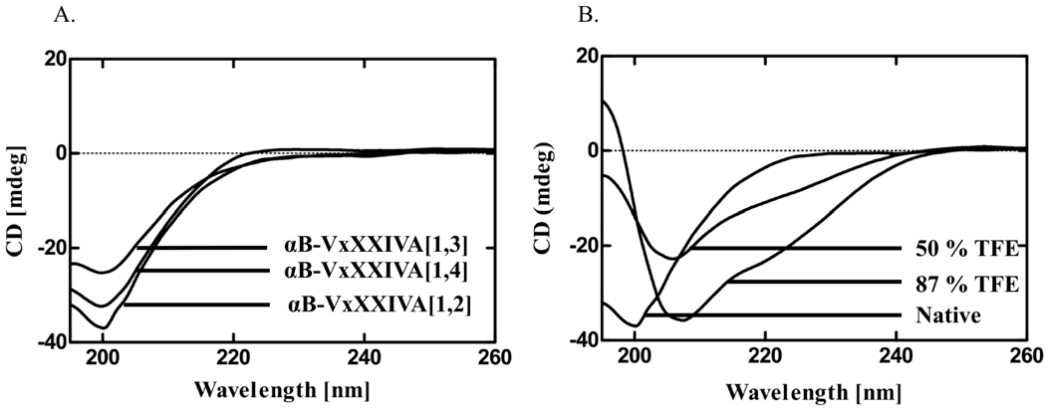
CD spectra αB-Conotoxin VxXXIVA isomers. (**A**) Overlay of spectra in phosphate buffer. (**B**) CD spectrum of αB-VxXXIVA [1], [2] in the presence of 50 and 87% TFE. This isomer showed a propensity to adopt a partially helical conformation at high TFE concentrations as evident by the shift in the minimum towards 208 nm along with some ellipticity developing around 222 nm.

CD spectra were acquired on all threeαB-VxXXIVA isomers in phosphate buffer. All peptide isomers exhibited minima at around 200 nm (Fig. 7A), indicative of a random coil conformation with no α-helical and β-sheet content, and consistent with our NMR results. As TFE is known to stabilize the α-helical structure in proteins and peptides [19], CD spectra of one of the isomers (αB-VxXXIVA [1], [2]) were recorded in increasing concentrations of TFE. Upon addition of 50–85% TFE, αB-VxXXIVA [1], [2] showed slightly increased ordered structure, as evident by the shift in the minimum towards 208 nm, with some ellipticity also developing at 222 nm (Fig. 7B). The CD data were fitted using three algorithms (CDSSTR, CONTINLL, and SELCON) in CDPro [16]. The outputs obtained from all three algorithms gave very similar values and indicated that the αB-VxXXIVA [1], [2] isomer in the presence of 87% TFE had ∼ 42% α-helix, ∼ 8% β-strand and ∼ 50% unordered structure (including turns), whereas, in the absence of TFE it had ∼ 7% α-helix, ∼ 31% β-strand and ∼ 62% unordered structure.

## Discussion

Conotoxins are a highly specialized set of disulfide-bonded peptides that are structurally and functionally diverse. Despite this diversity, toxins identified to date may be grouped into approximately 17 gene superfamilies based on conservation of the signal sequence. Within these gene superfamilies, the mature peptides adopt one of 23 patterns of arrangement of cysteine residues. Pharmacological targets within a gene superfamily may differ. For example, in the A superfamily, there are both paralytic and excitotoxic peptides [20].

It is very likely that the previously described superfamilies and Cys frameworks represent only a small fraction of the total chemical space of conotoxins. *C. vexillum* inhabits waters up to 70 m deep in Hainan province of the South China Sea and feeds on eunicid worms. Here, we describe the discovery and characterization from this species of αB-VxXXIVA, a peptide that differs in substantial aspects from previously-reported conotoxins.

The clone for αB-VxXXIVA was obtained from random sequencing of a cDNA library prepared from venom ducts. The signal sequence of αB-VxXXIVA does not align well with the signal sequence of other known conotoxins. Conservation of the signal sequence has previously been exploited as a means of cloning novel conotoxins from different species of cone snails [21]. The unique signal sequence of αB-VxXXIVA explains why this novel conotoxin has not been detected previously with screening primers designed to recognize known gene superfamilies. The discovery of αB-VxXXIVA expands the known complexity of this group of ion channel- and receptor-targeted ligands. Interestingly, the precursor for αB-VxXXIVA is unique among conotoxins in that it lacks a pro region. The pro region of disulfide-bonded peptides has been shown to facilitate oxidative folding [22]. Consequently, the pro region of conotoxins was originally proposed as a means by which these peptides could fold into the same three-dimensional scaffold with identical disulfide connectivity [23]. However, evidence from studies with the two-disulfide α-conotoxin GI and three disulfide ω-conotoxin MVIIA [24] indicates that the propeptide sequence does not necessarily contribute directly to folding thermodynamics but rather plays a facilitative role when folding is catalyzed by a disulfide isomerase [25]. The pro domain has also been implicated in the secretory pathway of hydrophobic O-superfamily conotoxins [26]. Apparently, such a mechanism is not necessary for the more hydrophilic αB-VxXXIVA.

The mature αB-VxXXIVA toxin is 40 amino acid residues in length and has a previously unreported arrangement of four Cys residues, C-CC-C. We synthesized the three possible disulfide isomers (Fig. 2) and assessed their activity at nAChRs. There are no reported examples of conotoxins that contain a vicinal disulfide bridge, and in the present case, the isomer that was synthesized with linkage between the adjacent second and third Cys residues was inactive. Both of the other two possible disulfide connectivities, αB-VxXXIVA [1], [2] with a disulfide connectivity I–II, III–IV, and αB-VxXXIVA [1], [3] with I–III, II–IV, blocked αα9α10 nAChRs, with the I–II, III–IV connectivity being 2-fold more active than the I–III, II–IV form.

There is precedent for conotoxins that selectively block the α9α10 over other nAChR subtypes. α-Conotoxin Vc1.1 from *C. victoriae* and α-conotoxin RgIA from *C. regius* block the α9α10 nAChR with IC_50_ values of 5 and 19 nM, respectively [27]. Vc1.1 also blocks α6/α3β2β3 and α3β4 nAChRs with IC_50_ values of 140 and 4200 nM, respectively. Both α-CTx Vc1.1 and α-CTx RgIA were subsequently found to activate GABA_B_ receptors [27], [28], [29]. In addition, other conotoxins that block nAChRs have also been reported to block voltage-gated ion channels including sodium and potassium channels [30], [31]. The IC_50_ values for the αB-VxXXIVA isomers against α9α10 nAChRS are in the micromolar range. It is therefore possible that these peptides, in addition to blocking nAChRs, will subsequently be found to act on other ligand- or voltage-gated ion channels.

Although cone snails hunt fish, molluscs and worms, worms are the most common prey. The nAChR subunits from these polychaete marine worms have not been cloned; however, it is of note that αB-VxXXIVA preferentially targets the α9α10 subtype of nAChR. The α9 subunit is a member of the nAChR family although it is more distantly related; indeed it appears to be the closest subunit to the ancestor that gave rise to the nAChR family [32]. Thus, it is tempting to speculate that, among *Conus*, the worm-hunting species may be particularly likely to produce toxins that target α9 receptors.

The α9 subunit is also of increasing interest in biomedicine. Conotoxins that target the α9 nAChR have been shown to be analgesic [10], [27] and to accelerate the recovery of function after nerve injury, possibly through immune-mediated mechanisms [33], [34]. In addition, small molecule antagonists of α9α10 nAChRs are analgesic in models of neuropathic pain [35], [36].

The α9α10 receptor is present in keratinocytes and is implicated in the pathophysiology of wound healing [37]. Recently it has been shown that the α9 subunit is overexpressed in breast cancer tissue. α9 antagonists reduce tumour growth [38], [39]. Moreover, variants of the α9 subunit affect transformation and proliferation of bronchial cells [1], [40]. Thus, novel antagonists of the α9α10 nAChR are not only of value to structure/function analysis of this receptor subtype but may also help inform development of novel therapeutics.

The αB-VxXXIVA toxins are atypical among disulfide-bridged conotoxins in showing largely disordered structures in aqueous solution over a range of temperature and pH values. While unusual, this is consistent with structure predictions that show no significant ordered secondary structure for this amino acid sequence (Fig. S5); presumably this is also why the addition of a helix-stabilizing co-solvent like TFE did not induce significant helical structure in αB-VxXXIVA (Fig. 7). There are, however, precedents for disulfide-bridged conotoxins with poorly ordered structures and potent biological activity. Synthetic α−AuIB, for example, formed both a globular (native) isomer and a ribbon isomer upon oxidative refolding, and the ribbon isomer, although having a less well-defined structure, had approximately 10 times greater potency than the native peptide on nACh-evoked currents in rat parasympathetic neurons [41]. More recently, three different disulfide-bridge isomers of the μ-conotoxin PIIIA, which contains three disulfides, were found to block the skeletal muscle voltage-gated sodium channel Na_V_1.4 with similar, yet distinct potencies [42] even though one of them was disordered and gave a poorly dispersed ^1^H NMR spectrum akin to those observed for all three αB-VxXXIVA disulfide isomers.

The concept of intrinsically disordered proteins is well established now [43], although it is quite unusual to find a conotoxin containing two disulfide bridges that displays these properties, as in the case of αB-VxXXIVA. It is believed that most intrinsically disordered proteins adopt a more ordered structure upon binding to their physiological targets [44], although evidence is emerging that this is not always the case. It remains to be seen if αB-VxXXIVA becomes more ordered upon binding to α9α10 nAChR. This might be assessed by studying the interaction of ACh-binding proteins engineered to resemble the α9α10 nAChR [45] and/or by creating conformationally constrained analogues of αB-VxXXIVA.

## Supporting Information

Figure S1 **The** ***amide and aromatic region*** **of ^1^H** ***NMR spectra*** **of αB-VxXXIVA** [**1]**, **[2]** **isomer at pH 5.8 and 7.0 in 20 mM phosphate buffer, acquired on a Varian 600 MHz NMR spectrometer at 22°C.** Note that fewer amide resonances are observed at pH 7.0 because some are in rapid to intermediate exchange with solvent water at this pH.
(TIFF)

Figure S2 **The** ***amide and aromatic region*** **of ^1^H** ***NMR spectra*** **of αB-VxXXIVA** [**1]**, **[3]** **isomer at pH 5.8 and 7.0 in 20 mM phosphate buffer, acquired on a Varian 600 MHz NMR spectrometer at 22°C.** Note that fewer amide resonances are observed at pH 7.0 because some are in rapid to intermediate exchange with solvent water at this pH.
(TIFF)

Figure S3 **The** ***amide and aromatic region*** **of ^1^H** ***NMR spectra*** **of αB-VxXXIVA** [**1]**, **[4]** **isomer at pH 5.8 and 7.0 in 20 mM phosphate buffer, acquired on a Varian 600 MHz NMR spectrometer at 22°C.** Note that fewer amide resonances are observed at pH 7.0 because some are in rapid to intermediate exchange with solvent water at this pH.
(TIFF)

Figure S4 **^1^H NMR spectra of αB-VxXXIVA** [**1]**, **[2]** **in the presence and absence of CaCl_2,_ in 90% H_2_O/10% ^2^H_2_O at pH 5.5, acquired on a Varian 600 MHz NMR spectrometer at 22°C.**
(TIFF)

Figure S5 **Secondary structure prediction of αB-VxXXIVA isomer, using the** ***PSIPRED*** **protein structure prediction** ***server (*** http://bioinf.cs.ucl.ac.uk/psipred/ ***).***
(TIFF)

Table S1 **Alignment of protein precursor sequences of** ***Conus*** **gene superfamilies from** **Table 1** **.**
(DOC)

Table S2 **Alignment of mature toxin sequences of nAChR targeted conotoxin superfamilies from** **Table 2** **.**
(DOC)
