# Supplementary figures and images for: A Novel Inhibitor of α9α10 Nicotinic Acetylcholine Receptors from Conus vexillum Delineates a New Conotoxin Superfamily
Source: PLoS One. 2013 Jan 30;8(1):e54648. doi: 10.1371/journal.pone.0054648 (PMC3559828; doi:10.1371/journal.pone.0054648)

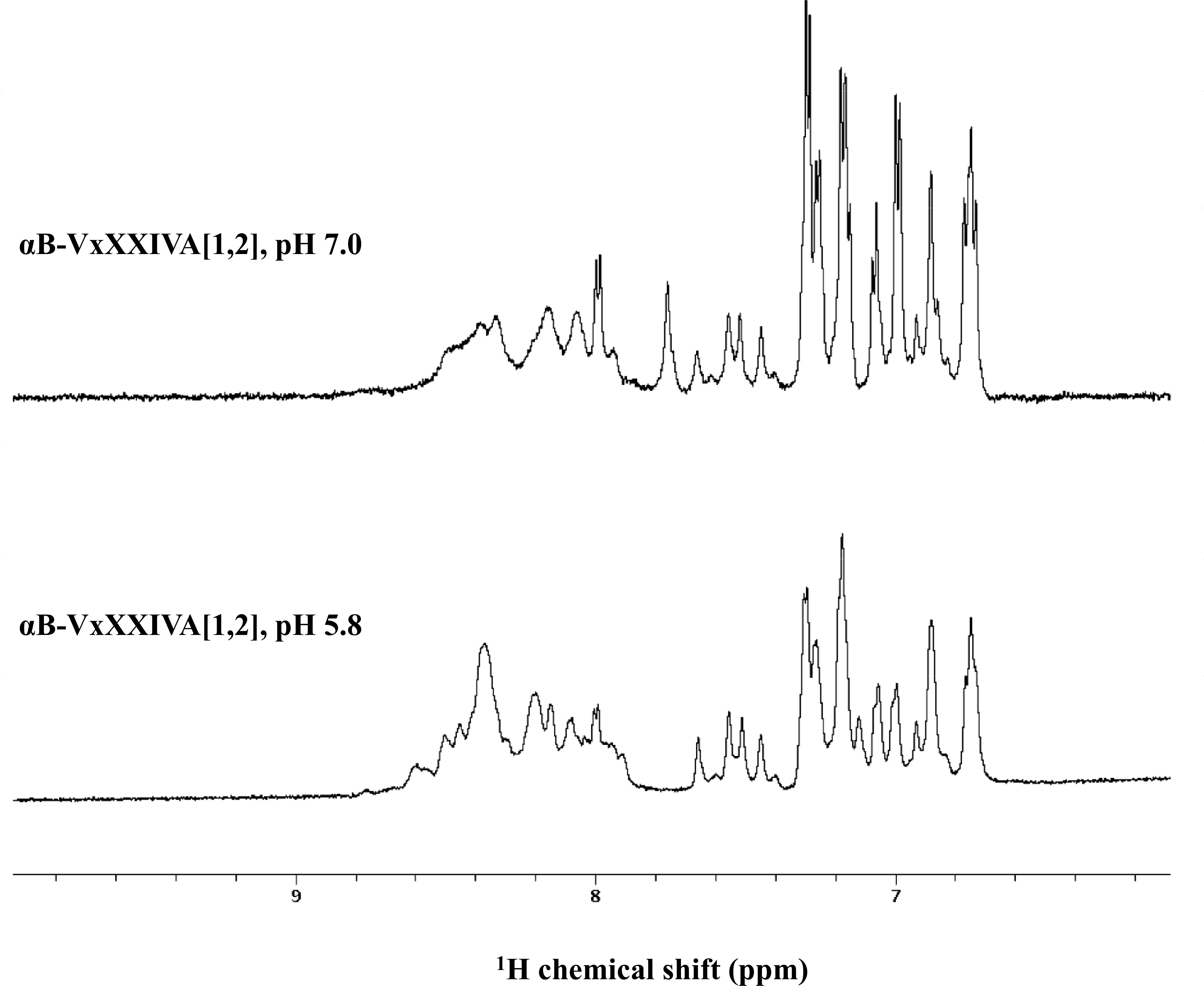

Supplement: Figure S1 — The amide and aromatic region of 1H NMR spectra of αB-VxXXIVA [1] , [2] isomer at pH 5.8 and 7.0 in 20 mM phosphate buffer, acquired on a Varian 600 MHz NMR spectrometer at 22°C. Note that fewer amide resonances are observed at pH 7.0 because some are in rapid to intermediate exchange with solvent water at this pH. (TIFF) [file pone.0054648.s001.tiff]

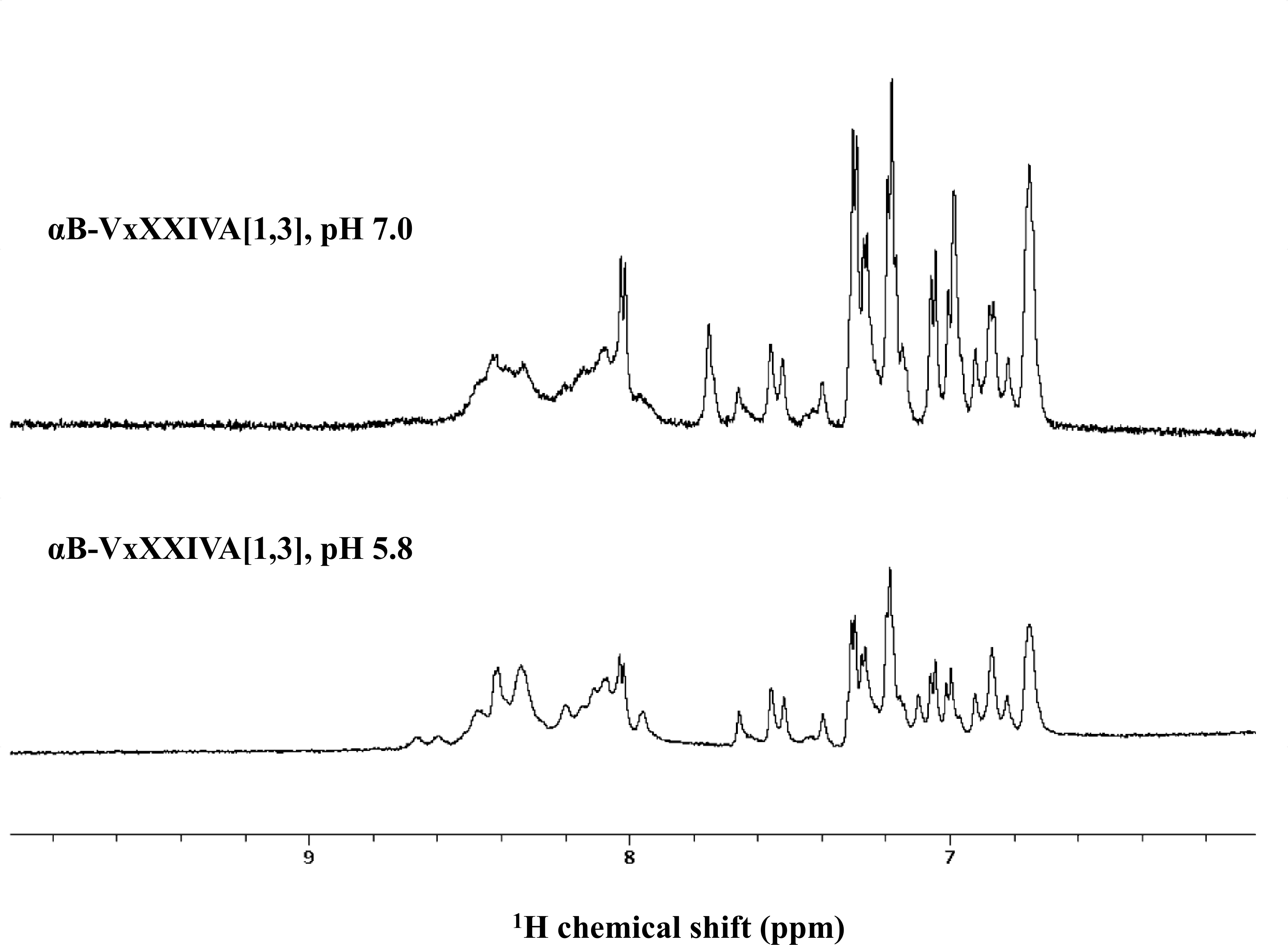

Supplement: Figure S2 — The amide and aromatic region of 1H NMR spectra of αB-VxXXIVA [1] , [3] isomer at pH 5.8 and 7.0 in 20 mM phosphate buffer, acquired on a Varian 600 MHz NMR spectrometer at 22°C. Note that fewer amide resonances are observed at pH 7.0 because some are in rapid to intermediate exchange with solvent water at this pH. (TIFF) [file pone.0054648.s002.tiff]

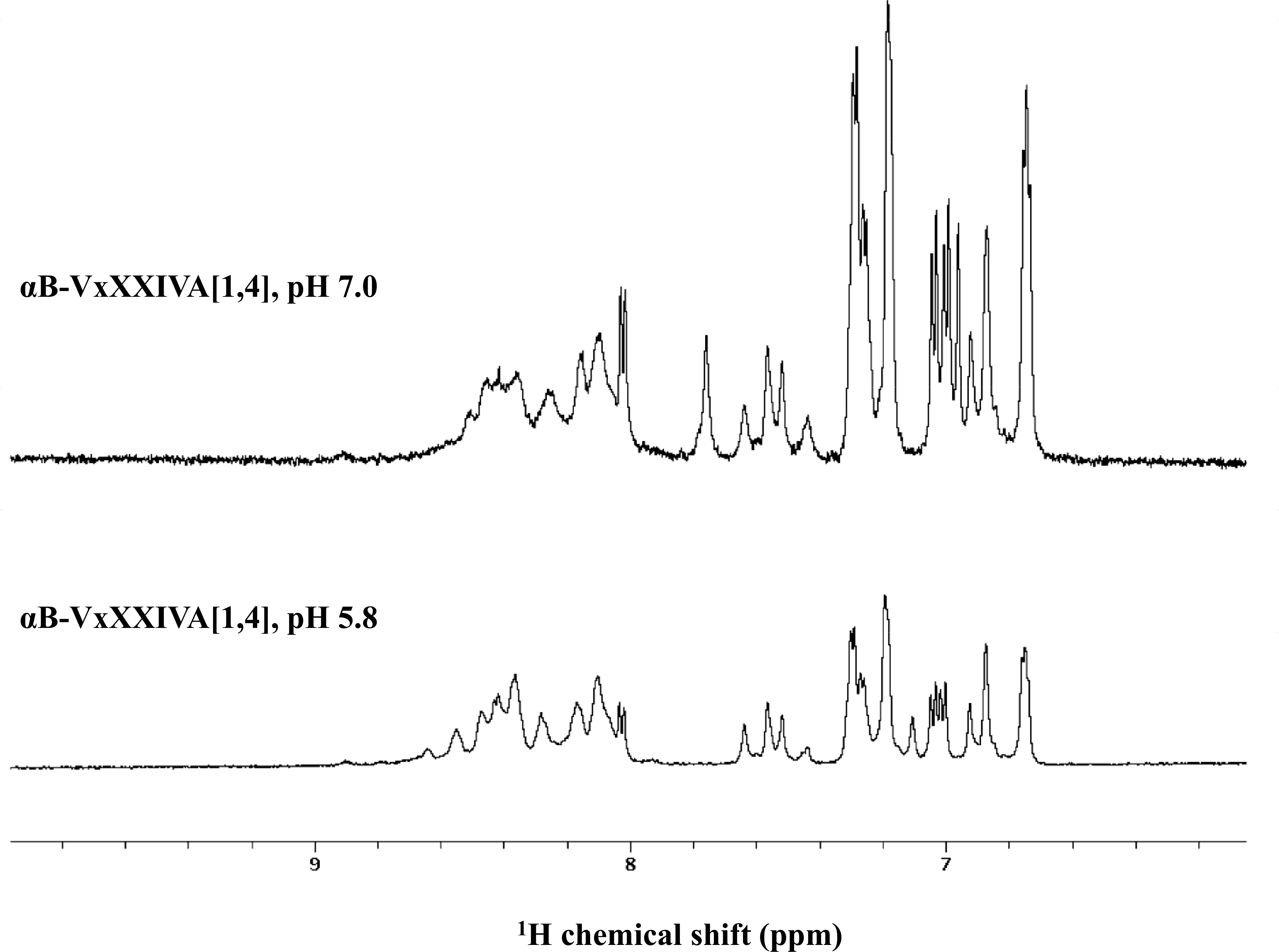

Supplement: Figure S3 — The amide and aromatic region of 1H NMR spectra of αB-VxXXIVA [1] , [4] isomer at pH 5.8 and 7.0 in 20 mM phosphate buffer, acquired on a Varian 600 MHz NMR spectrometer at 22°C. Note that fewer amide resonances are observed at pH 7.0 because some are in rapid to intermediate exchange with solvent water at this pH. (TIFF) [file pone.0054648.s003.tiff]

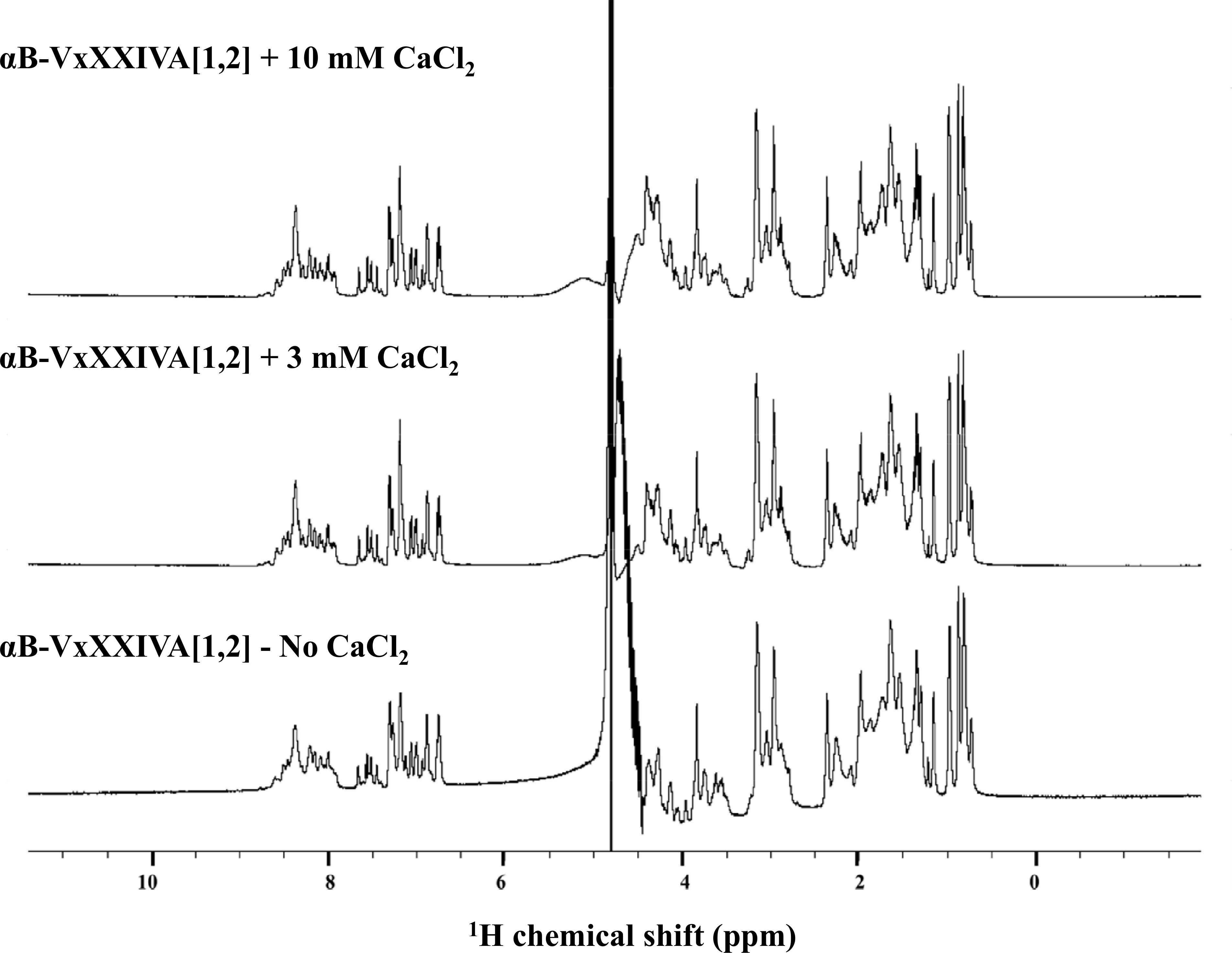

Supplement: Figure S4 — 1H NMR spectra of αB-VxXXIVA [1] , [2] in the presence and absence of CaCl2, in 90% H2O/10% 2H2O at pH 5.5, acquired on a Varian 600 MHz NMR spectrometer at 22°C. (TIFF) [file pone.0054648.s004.tiff]

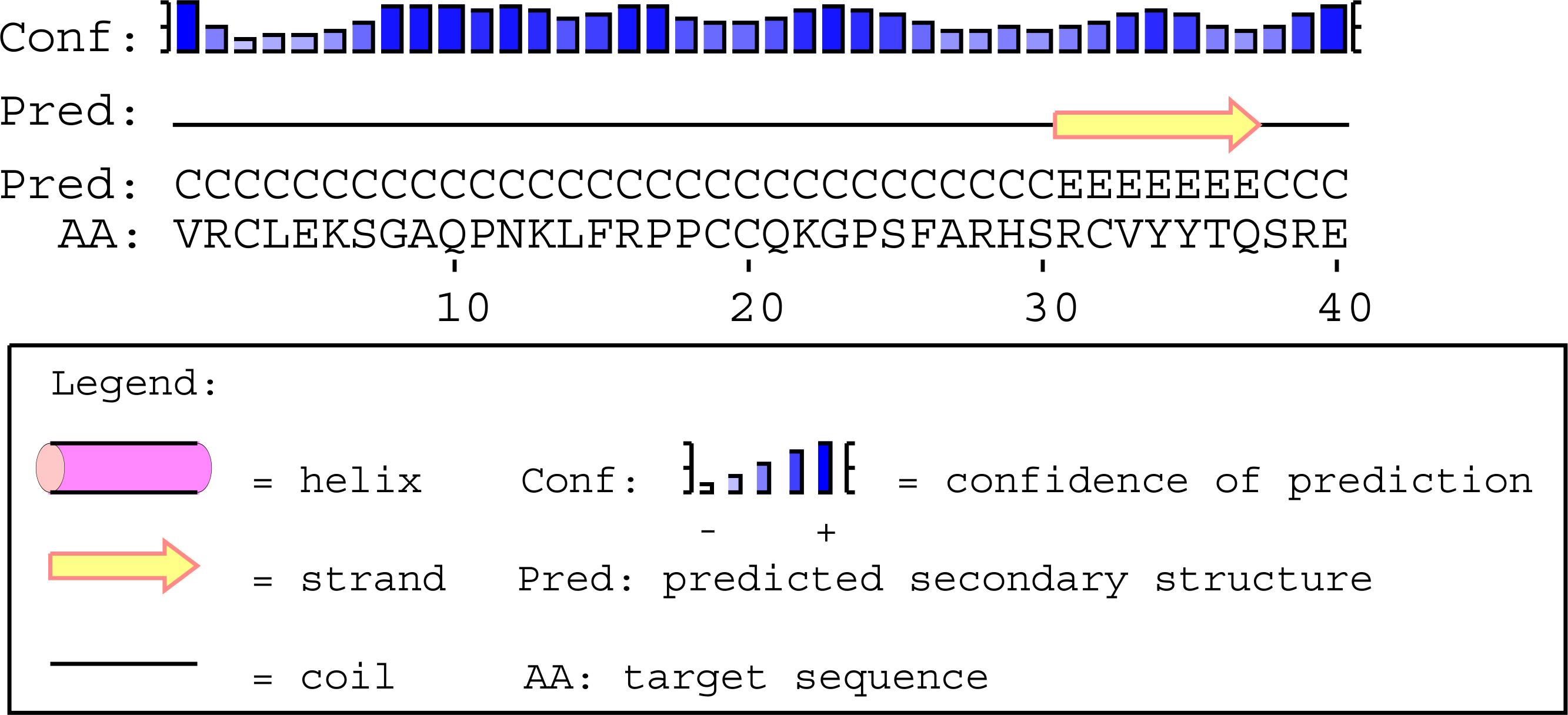

Supplement: Figure S5 — Secondary structure prediction of αB-VxXXIVA isomer, using the PSIPRED protein structure prediction server ( http://bioinf.cs.ucl.ac.uk/psipred/ ). (TIFF) [file pone.0054648.s005.tiff]
