# Supplementary material for: A Novel Inhibitor of α9α10 Nicotinic Acetylcholine Receptors from Conus vexillum Delineates a New Conotoxin Superfamily
Source: PLoS One. 2013 Jan 30;8(1):e54648. doi: 10.1371/journal.pone.0054648 (PMC3559828; doi:10.1371/journal.pone.0054648)
Supplement: Table S1 — Alignment of protein precursor sequences of Conus gene superfamilies from Table 1 . (DOC) [file pone.0054648.s006.doc]

| **Superfamily** | | | **Peptide** | **Signal peptide** | **N-terminal pro-regions** |
| --- | --- | --- | --- | --- | --- |
|  | B  A  D  I_1_  I_2_  I_3_  J  K  L  M  O_1_  O_2_  O_3_  P  S  T  V  Y | **α**B-VxXXIVA  **α**-LtIA  **α**D-VxXXB  ArXIA  BeTX  Ca11.3  Fe14.1  im23a  C14.1a  ψ-PrIIIE  SO3  BeB54  CaFr179  GmIXA  **α**S-GVIIIA  VcVB  ViXVA  CaXVIIA | | ***M***ET***L***TL***L***WRASSSCL***L***VVLSHSLLRLLG  ***M***GMRMMFIMFM***L***VVLAT***T***VVTFTS  ***M***PK***L***AVV***LLVLL***I***L***P***L***SYFDAAGG  ***M***K***L***CATF***LLVL***VT***L***P***L***V***T***G  ***M***MFRVTSVGC***LLL***VIVFLNLVVPTSA  ***M***K***L***VLAIVVI***L***M***LL***S***L***S***T***GA  ***M***PSVRSVTCCC***LL***W***M***MLSVQLVTP  ***M***IMRMT***L***T***L***FV***L***VV***M***TAASASG  ***M***NVTVMF***LV***L***LLL***T***M***P***LT***DG  ***M***SK***L***GV***LL***TIC***LLL***FPI***T***A  ***M***K***L***TCMVIVAV***LLL***TACQLIT***A***  ***M***EK***L***TI***LLLV***AAV***LM***STQALI  ***M***SG***L***GIMV***L***T***LLLL***VFMEA  ***M***H***L***SLARSA***VL***M***LL***L***L***FALGNFVVVQS  ***M***MSKMGAMF***VLLLL***FT***L***AS  VI***LL***LLIASAPSVDA  ***M***MPVIL***LLL***LS***L***AIRCADG  ***M***QKATV***LLL***A***LLLL***LP***L***STA | DRALDAMNA***AA***SNKAS***R***LIALAVR  ***Q***A***VQGD***W***R***GNR***LAR***D***L***QRGGR  EKSSERSL***S***G***A***IL***R***GV***R***  EMSDNHASRS***A***T***A***L***R***D***R***LLSPK  GSPGTA***Q***L***S***GHRTA***R***  DALTEAK***R***  FNIRATNGGELFGPVQ***R***DAGNVLD***H***GFQRR***R***  LP***V***D***GDQ***PADRPVE***R***MQDNISSEQ***H***PFFEK***R***  DDSR***G***T***Q***KHRT***L***RSKTKLSMSTR  ***Q***SDGEKRQQ***A***KINFLS.R  SHQDAGEKQ***A***TQRDAINVRRRRSLAR***R***  GLITR***D***VDNGQ***L***TDNR***R***NLQTEWNPLSLFMSRR  SLQE***GD***VQARKTRLKSDFYRALARDD***R***  ***Q***PKTK***D***DVPL***A***PLHDNAKSALQHLNQ***R***  KA***VQGD***SDPS***A***SLLTGDKNHDLPVKR  ***Q***DAE***G***S***Q***ED***AA***QREVDIATR |

| **Super family** | | | **Peptide** | | **Mature peptide** | **C-terminal pro-regions** |
| --- | --- | --- | --- | --- | --- | --- |
|  | B  A  D  I_1_  I_2_  I_3_  J  K  L  M  O_1_  O_2_  O_3_  P  S  T  V  Y | **α**B-VxXXIVA  **α**-LtIA  **α**D-VxXXB  ArXIA  BeTX  Ca11.3  Fe14.1  im23a  C14.1a  ψ-PrIIIE  SO3  BeB54  CaFr179  GmIXA  **α**S-GVIIIA  VcVB  ViXVA  CaXVIIA | | --VR-***C***LEK---S--***G***AP------NKLFRPP----***CC***QK***G***PSFARHS-***R***-***C***VYYTQSRE  ---------------***G***-***CC***ARAA-----***C***AGIHQEL***C***G***GG***R------------------  DDESE***C***IINTRDSPW***G***R***CC***R-----TRM***CG***SM---***CC***PRNGCTCVYHW***R***RGHG***C***SCPG-  ---RT***C***SRR***G***-----HR***C***IR***D***----***S***Q***CCG***GM---***CC***Q***G***NR--CFVAI***R***R***C***FHLPF---  -----***C***RAE***G***-----TY***C***EN***D***----***S***Q***CC***LNE---***CC***W***GGC***G-HP***C***--***R***HP--------  --ASI***C***YG***TG***-----***G***R***C***TK***D***----KH***CCG***WL---***CC***G***G***PSVG--***C***VVSVAP-***C***K----  -------SP***G***---------------***S***TI***C***KMA---***C***RT***G***NGHKY----PF***C***N-***C***R----  --IPY***C***GQ***TG***-----AE***C***YSWCIKQDLSKDW----***CC***DFVKDI—RMNPPADK-***C***P----  ----------------D***C***PPW-------***C***PTS—-H***C***NA***G***T***C***------------------  -AARC***CT***YH***G***------S***C***LK------EK***C***RRK—-Y***CC***-***G***R--------------------  -----***C***KAA***G***-----KP***C***-------***S***RIAYN----***CC***T***G***S***C***RSGK***C***G------------  ----KS***T***AESWWE—GECKGW-----SVY***C***SWDW-E***CC***S***G***E***C***TRYY***C***ELW----------  ----TV***T***EE--------***C***-------EED***C***EDEEKH***CC***NTNNG-PS***C***ARL-***C***FG------  ----S***C***NNS--------***C***QS-----HSD***C***ASHC-I***C***TFR***GC***GAVNG-------------  ----G***CT***R***T***CGGPK---***C***TGT-----CT***C***TNSSK-***C***GCRYNVHPSGWGCG***C***A-***C***SG---  -----***C***CQ***T***FYW-----------------------***CC***GQ***G***K-----------------  ----D***CT***TCAG--------------EE***CCG***RCT—-***C***PW***G***DN----***C***SCIEWGK-----  -----***C***GG***TG***D-----S***C***NEPAG—E-L***CC***RRLK—-***C***VNSR-----***C***CPTTDG-***C***---- | | ***GKR***SKLQEFFRQR  ***GKR***DVVSSSMAV |
