# Supplementary material for: A Novel Inhibitor of α9α10 Nicotinic Acetylcholine Receptors from Conus vexillum Delineates a New Conotoxin Superfamily
Source: PLoS One. 2013 Jan 30;8(1):e54648. doi: 10.1371/journal.pone.0054648 (PMC3559828; doi:10.1371/journal.pone.0054648)
Supplement: Table S2 — Alignment of mature toxin sequences of nAChR targeted conotoxin superfamilies from Table 2 . (DOC) [file pone.0054648.s007.doc]

| **Peptide** | **Mature peptide** | |
| --- | --- | --- |
| **α**B-VxXXIVA  **α**-AuIB  **α**A-OIVB  **α**C-PrXA  **α**D-VxXIIA  **α**S-RVIIIA  ψ-PIIIE | VRCLEKS***G***AQPNKLFRPP***CC***QK--***GP***S--***F***--ARH***S***-R***C***VYYTQSRE^------------  -------***G***----------***CC***SY--P***PC***--***F***--ATNP-D***C***#--------------------  ------------------***CC***GVONAA***C***--------P-O***C***V***C***NKTC***G***#-------------  -------TYGIYDA***K***POFS***C***AGLR***G***G***C***------VLPONLROKFKE#--------------  --DVQD***C***QVSTOGS***K***WGR***CC***LN--RV***C***GP-MC***C***PA***S***-H***C***Y***C***VYHR***G***-RGH***GCSC***^-----  -----K***C***NFDKCKGTGVYN***C***GX-SCS***C***X-----GLH-***SC***R***C***TYNI***G***SMKS***GC***A***C***ICTYY^  ---------------HOO***CC***LY--***G***K***C***RRYOG***C***S-***S***A***SC***-***C***QR#---------------- |  |
